# Pain Control, Acceptance and Adjustment to the Disease among Patients with Ovarian, Endometrial and Breast Cancer in Poland

**DOI:** 10.3390/ijerph182212148

**Published:** 2021-11-19

**Authors:** Aleksandra Czerw, Urszula Religioni, Katarzyna Sygit, Agnieszka Nieradko-Heluszko, Dominika Mękal, Olga Partyka, Marcin Mikos, Mateusz Eid, Łukasz Strzępek, Tomasz Banaś

**Affiliations:** 1 Department of Health Economics and Medical Law, Medical University of Warsaw, 02-091 Warsaw, Poland; 2 Department of Economic and System Analyses, National Institute of Public Health NIH-National Research Institute, 00-791 Warsaw, Poland; opartyka@pzh.gov.pl; 3 Collegium of Business Administration, Warsaw School of Economics, 02-513 Warsaw, Poland; urszula.religioni@gmail.com; 4 Faculty of Health Sciences, Calisia University, 62-800 Kalisz, Poland; ksygit@poczta.onet.pl; 5 Subdepartment of Social Medicine and Public Health, Department of Social Medicine, Pomeranian Medical University in Szczecin, 70-103 Szczecin, Poland; agnieszka.nieradko.heluszko@pum.edu.pl; 6 Department of Cancer Prevention, Medical University of Warsaw, 02-091 Warsaw, Poland; dmekal@wum.edu.pl; 7 Faculty of Medicine and Health Sciences, Andrzej Frycz Modrzewski Krakow University, 30-705 Krakow, Poland; mikos@ziz.com.pl; 8 Faculty of Health Sciences, Medical University of Warsaw, 02-091 Warsaw, Poland; mateusz.eid@gmail.com; 9 Department of General Surgery, Regional Public Hospital in Bochnia, 32-700 Bochnia, Poland; strzepeklukasz@wp.pl; 10 Department of Gynecology and Obstetrics, Jagiellonian University Medical College, 31-501 Krakow, Poland; tbanas@mp.pl

**Keywords:** breast cancer, disease acceptance, endometrial cancer, ovarian cancer, pain

## Abstract

Background: Breast, ovarian, and endometrial cancer are among the most common causes of morbidity and mortality of women in Poland. In 2016, breast cancer was the most common cause of morbidity and the second leading cause of cancer deaths in women, endometrial cancer was the third most common cause of morbidity and the seventh leading cause of death, and ovarian cancer was the fifth most common cause of morbidity and the fourth leading cause of cancer deaths in women. The aim of the study was to assess the strategy of pain control, acceptance of the cancer and adjustment to life with disease in women with ovarian cancer, endometrial and breast cancer. This study shows how level of pain control, acceptance, and adjustment can differ among patients with the three kinds of cancer and which factors have the most influence on patients’ adjustment to the disease. Methods: The study was carried out with 481 patients diagnosed with ovarian cancer, endometrial and breast cancer. In the study BPCQ, CSQ, AIS and Mini-MAC questionnaires were used. Results: In the BPCQ questionnaire the highest result was acquired in the scope of the impact of doctors (M = 16.45, SD = 4.30), differentiated by cancer location and socio-economic variables. In the CSQ test, the highest result was achieved by praying/hoping, differentiated by cancer location and socio-economic variables. The average AIS acceptance score was M = 27.48 (SD = 7.68). The highest result of the Mini-Mac scale was obtained by patients in the area of fighting spirit (M = 22.94, SD = 3.62), and these results depended on socio-economic and treatment-related variables but were not differentiated by cancer location. Conclusions: Patients attribute the highest importance in the disease to the influence of physicians, praying/hoping, and fighting spirit. The awareness of the pain management strategies of patients with cancer allows appropriate psychological support to be designed for specific groups of patients.

## 1. Introduction

Malignant epithelial breast, ovarian and endometrial cancers are among the most common causes of morbidity and mortality among women in Poland. In 2016, breast cancer was the most common cause of cancer-related morbidity among women (accounting for 22.8% of cases), endometrial cancer was in the third position (responsible for 7.7% of cancer cases among women), and ovarian cancer was the fifth most common cause of cancer-related morbidity (4.6% of cases) [1].

In terms of cancer mortality, after lung cancer, breast cancer is the second most common cause of female deaths in Poland (responsible for 14.5% of deaths), ovarian cancer is the fourth (5.9%), and endometrial cancer the seventh (3.6% deaths) [1].

Pain can be defined as subjective unpleasant sensory experience in response to tissue damage. This experience is influenced by many factors: biological, social, and psychological [2]. Control of pain can improve patients’ quality of life, even when the prognosis is bad. The main objective of pain control in cancer patients is to minimalize the adverse effects of treatment and to improve comfort [3].

Understanding quality of life is essential for proper treatment and patients’ rehabilitation. The World Health Organization defines quality of life as “An individual’s perception of their position in life in the context of the culture in which they live and in relation to their goals, expectations, standards and concerns” [4]. However, there is still an ongoing debate on methodological standards defining measures of quality of life.

It has been shown that both cancer and the therapy process used have a significant impact on the quality of life. A worse mood and the limitations resulting from cancer cause social dysfunction in patients. Very often patients limit their professional activity, causing a deterioration of their social and living conditions; they restrict social life to the circle of close family and friends, often resulting in the need for help from other people [5]. Women with diagnosed ovarian cancer are characterized by a certain specificity of problems affecting their quality of life. Diagnosis and treatment of this disease are extremely overwhelming and constitute a significant adjustment challenge. These are primarily aspects concerning the image of one’s body, the perception of oneself as a woman, sexual functioning, infertility (especially important for younger women), and premature menopause [6,7]. The quality of life assessed by patients is influenced by the approach to cancer, pain coping strategies, acceptance, and adjustment to the disease.

The aim of the study was to assess the location of pain control, strategies of coping with pain, the level of acceptance of cancer, and adjustment to life with disease of women with ovarian, endometrial and breast cancer. The study looked for relationships between socio-economic factors (sex, age, education, occupational status, income, place of residence) and the fact of diagnosis of metastases, the type of treatment used, and the results obtained in psychometric tests.

## 2. Materials and Methods

The study was carried out between 2013 and 2018 out of 481 patients aged 21–96 (M = 56.66; SD = 12.87) diagnosed with ovarian cancer aged 21–82 (M = 53.22; SD = 13.03), endometrial cancer aged 26–83 (M = 56.23; SD = 12.94), and breast cancer aged 30–96 (M = 60.03; SD = 11.84), who at least one month earlier had ended active oncological treatment and remained under outpatient control at the Oncology Center-Maria Skłodowska-Curie Institute in Warsaw. The socio-economic characteristics of the sample are presented in Table 1. 

The research tool was a questionnaire with metric questions and four psychometric tests. The Paper and Pencil Interview (PAPI) technique was applied

1. The Beliefs about Pain Control Questionnaire (BPCQ), intended to test people experiencing pain. The BPCQ test contains 13 statements that make up three factors that evaluate the strength of individual beliefs regarding control of pain: in person (internal factors), through the impact of physicians (other forces), or through random events [8,9]. The higher the scores the more power in controlling the pain is ascribed to the factor.
2. The Pain Coping Strategies Questionnaire (CSQ) was designed to examine people who express pain. The questionnaire contains 42 theses. The mechanisms of coping with pain assessed in the questionnaire demonstrate six cognitive and one behavioral strategy for coping with pain, and they are components of three factors: catastrophizing and seeking hope, cognitive coping, distraction and shifting to substitute actions [9,10]. The higher the score the more meaning is ascribed to the relevant strategy of coping.
3. Approval Illness Scale (AIS) is used to measure the level of adjustment to the disease. The scale of disease acceptance contains eight statements describing the negative consequences of ill health, based on which possible results for every one of the respondents in the level of acceptance of the disease in the range from 8 to 40. A higher score means better adjustment to the disease and less psychological discomfort. A lower result indicates the intensity of negative emotions related to the disease, which translates into a lower acceptance of the disease [9].
4. Mental Adjustment to Cancer (Mini-MAC), tests the level of mental adjustment to cancer. Mini-Mac is used to measure four strategies of coping with cancer: anxiety, fighting spirit, helplessness–hopelessness, and positive reevaluation. According to the assumptions of the questionnaire, anxiety and helplessness–hopelessness are part of a destructive style of coping with the disease, fighting spirit and positive reevaluation are part of a constructive style of coping with cancer [9].

The obtained results of the psychometric tests were statistically analyzed with the use of the Kruskal–Wallis H test for comparison between three groups of participants regarding each dependent variable, the Mann–Whitney U test for comparison between two groups of participants regarding each dependent variable, and the Spearman’s correlation (in case of age). Also, the Mann–Whitney U test was used as follow-up test when the Kruskal–Wallis H test was statistically significant. The adopted level of statistical significance was *p* < 0.05.

The results of the tests were compared with the socio-economic characteristics of the respondents: their gender, level of education, age, the size of the place of residence and income per member of the household, professional status, the fact of diagnosis of metastases, and the type of treatment to which the patients were subjected.

## 3. Results

The study involved 481 women aged 21–96 (M = 56.66, SD = 12.87). In 193 cases (40.1%) breast cancer was diagnosed, in 172 cases (35.8%) ovarian cancer, and in 116 cases (24.1%) endometrial cancer.

### 3.1. Pain Control

For patients suffering from ovarian, endometrial and breast cancer, the highest average score in the BPCQ questionnaire was obtained on the influence of physicians (M = 16.45, SD = 4.30), and the lowest on random events (M = 15.25, SD = 4.46) (Table 2).

The results obtained were differentiated by the location of the neoplastic lesion. Significant differences were obtained in the area of the effect of physicians (*p* = 0.008) and random events (*p* = 0.001). The area of the influence of physicians was rated the highest by patients with breast cancer (M = 17.4, SD = 4.40), and the lowest by patients with ovarian cancer (M = 15.73, SD = 3.99). A similar relationship characterizes the area of random events, which was rated the highest by breast cancer patients (M = 16.22, SD = 4.32), and the lowest by patients with ovarian cancer (M = 14.41, SD = 4.49).

Pain control among the whole group of the studied patients was differentiated by all the socio-economic variables. There were statistically significant positive correlations between age of the patients and the location of pain control on the influence of physicians (ρ = 0.229, *p* < 0.01) and on random events (ρ = 0.145, *p* < 0.01), which averages that in both cases older patients attributed more importance to these areas. 

The level of education of patients influenced the results obtained in all areas of the BPCQ test, i.e., external factors χ^2^(2) = 16.21, *p* < 0.001, influence of physicians χ^2^(2) = 27.30, *p* < 0.001, and random events, χ^2^(2) = 22.85, *p* < 0.001.). Patients with education on the primary/vocational level obtained higher scores in each area of pain control than other patients, and women with higher education were characterized by the lowest average in each of the areas of the BPCQ test (Table 3).

Place of residence differentiated the patients on the point of assessing the impact of physicians on pain control, U = 23,680.50, *p* = 0.002. Women living in smaller towns up to 100,000 residents attributed a greater role in pain control to physicians (M = 17.01, SD = 4.01) than women living in cities over 100,000 residents (M = 15.72, SD = 4.54).

The level of income affected the average obtained in the area of the influence of physicians, U = 24,870.00, *p* = 0.011 and random events, U = 25,079.00, *p* = 0.016. In both cases women whose average monthly income per person in the household was lower (below PLN 1.500) were characterized by higher values in both these areas (for influence of physicians M = 17.05, SD = 4.19; for random events M = 15.77, SD = 4.37) compared to women whose income per person in the household was higher (for influence of physicians M = 15.94, SD = 4.32; for random events M = 14.80, SD = 4.48).

Professional status of the patients also affected the areas of the influence of physicians and random events (U = 17,517.00, *p* = 0.001 and U = 19,576.00, *p* = 0.009). Pensioners were characterized by higher averages both in the area of the influence of physicians (M = 17.29, SD = 4.35), as well as random events, than patients who were professionally active (M = 15.71, SD = 4.07 for the influence of physicians and M = 14.79, SD = 4.57 for random events).

The diagnosis of metastases influenced the area of internal pain control, U = 18,102.00, *p* = 0.013. This area was higher in women with no metastases (M = 16.19, SD = 5.03) than for patients with them (M = 14.98, SD = 5.81).

Chemotherapy and radiotherapeutic treatment did not affect pain control among the patients. Targeted treatment differentiated the area of the influence of physicians, U = 6372.00, *p* = 0.041, in which patients who were not treated with targeted therapy were characterized by a higher average (M = 16.57, SD = 4.27) than patients who were treated with targeted therapy (M = 14.94, SD = 4.39).

### 3.2. Strategies for Coping with Pain

In the CSQ test the highest average score for patients suffering from ovarian, endometrial and breast cancer was obtained by the area of praying/hoping (M = 21.47, SD = 8.89), then declaring coping (M = 21.39, SD = 9.21), and increased behavioral activity (M = 20.86, SD = 8.68). The smallest values are visible in the areas of reevaluation of pain sensations (M = 12.57, SD = 9.13) and catastrophizing (M = 11.67, SD = 8.04). The location of neoplastic lesions only affected the area of praying/hoping (*p* = 0.040). The highest average in this area belonged to patients with ovarian cancer (M = 22.73, SD = 7.87), and the lowest by patients with breast cancer (M = 20.24, SD = 9.11) (Table 4).

The age of patients influenced the average values obtained in the areas of ignoring pain sensations (ρ = 0.176, *p* = < 0.01), praying/hoping (ρ = 0.123, *p* < 0.01), declaring coping (ρ = 0.128, *p* < 0.01), and increased behavioral activity (ρ = 0.155, *p* < 0.01).

The education of patients differentiated the areas of reevaluation of pain sensations χ^2^(2) = 18.54, *p* < 0.001, ignoring sensations χ^2^(2) = 11.39, *p* < 0.001, and praying/hoping χ^2^(2) = 16.29, *p* < 0.001. For each of the areas, the highest averages were obtained by patients with primary education, and the lowest by patients with higher education (Table 5).

Place of residence differentiated only the area of praying/hoping, U = 22,337.50, *p* = 0.001. Women living in towns up to 100,000 inhabitants were distinguished by a higher average value of this area (M = 22.72, SD = 9.09) in comparison to women living in larger cities (M = 19.86, SD = 8.36).

Income had an impact on patients’ outcomes in the areas of ignoring pain, U = 479.00, *p* = 0.048 and praying/hoping, U = 481.00, *p* = 0.043. In both cases, patients with lower income per person in the household (up to PLN 1.500 per month) obtained higher results (M = 16.26, SD = 9.78 for ignoring pain and M = 22.30, SD = 9.16 for praying/hoping) than patients with higher income (M = 14.54, SD = 9.21 for ignoring pain and M = 20.76, SD = 8.60 for praying/hoping).

The professional status of patients suffering from ovarian, endometrial and breast cancer did not affect the strategies of coping with pain.

The presence of metastases differentiated the results obtained in the area of praying/hoping, U = 18,619.50, *p* = 0.038. Patients with metastases were found to have a higher result in this area (M = 22.62, SD = 8.87) than patients without metastases (M = 20.61, SD = 9.04). Chemotherapy treatment influenced patients’ results obtained in the area of distraction, U = 22.740, *p* = 0.021), with higher results obtained by women undergoing chemotherapy treatment (M = 20.87, SD = 8.75) than women not undergoing chemotherapy (M = 19.13, SD = 8.54).

Radiotherapeutic treatment differentiated the area of catastrophizing, U = 8720.50, *p* = 0.027. Women subjected to radiotherapy obtained a higher average in this area (M = 14.22, SD = 8.84) in comparison to women not treated with radiotherapy (M = 11.37, SD = 7.90).

Targeted treatment also influenced patients’ results obtained in the area of catastrophizing U = 5868.50, *p* = 0.007. The fact of targeted treatment increased the patients’ average value assigned to this area (M = 14.92, SD = 7.22) in comparison to the patients without targeted treatment (M = 11.41, SD = 8.05).

### 3.3. Acceptance of the Disease

The average AIS acceptance score obtained by patients suffering from ovarian, endometrial and breast cancer was M = 27.48 (SD = 7.68) (Table 6).

The average value of acceptance of the disease in the group of people diagnosed with breast cancer was the highest and it was M = 28.46 (SD = 7.98), for patients with endometrial cancer M = 26.99 (SD = 7.54), and among patients diagnosed with ovarian cancer it was M = 26.72 (SD = 7.35) (*p* < 0.05).

The age of patients had no statistically significantly correlation with the level of acceptance of the cancer, ρ(476) = 0.08, *p* > 0.05.

The average value of acceptance of the cancer in the group of patients with primary/vocational education was M = 25.88 (SD = 7.86), in the case of people with secondary education it was M = 27.57 (SD = 7.43), for people with higher education M = 28.35 (SD = 7.78), which was significantly higher than in the group of people with primary/vocational education U = 6921.50, *p* < 0.01.

For patients from towns with a population of up to 100,000 the average value of acceptance of the disease was M = 26.46 (SD = 8.06). This was a lower result than the average value acquired in the group of patients who lived in cities of over 100,000 inhabitants M = 28.86 (SD = 6.95), U = 23,626.00, *p* < 0.01.

The average value of acceptance of the disease for people with net income lower than PLN 1.500 per household member was M = 26.22 (SD = 7.69) and it was lower than the average value obtained in the group of people with income over PLN 1.500, M = 28.56 (SD = 7.52), U = 23,599.00, *p* < 0.01.

The average value of acceptance of the disease in the working group was M = 27.98 (SD = 7.07) and was close to the average value obtained in the group of pensioners amounting to M = 27.10 (SD = 8.25), U = 21,731.50, *p* > 0.05.

The average value of acceptance for people with metastases was M = 25.19 (SD = 7.66) and it was statistically significantly lower than the average value obtained in the group of people without metastases of M = 28.71 (SD = 7.51), U = 15,679.00, *p* < 0.001.

Similarly, the average value of acceptance of the disease in the group of patients who were subject to chemotherapy treatment was 5.66 (SD = 7.84). It was statistically significantly lower than the average value obtained for patients who were not subject to chemotherapy treatment M = 28.43 (SD = 7.43) (*p* < 0.001).

The average value of acceptance of the disease in the group of patients who underwent radiotherapy treatment was M = 25.84 (SD = 7.59) and it was close to the average value obtained in the group of people who were not treated with radiotherapy amounting to M = 27.67 (SD = 7.68), U = 9149.50, *p* > 0.05. Also, the average value of acceptance of the disease in the group of people who underwent targeted treatment was M = 25.72 (SD = 7.91) and it was close to the average value obtained in the group of people who did not use this treatment of M = 27.62 (SD = 7.65), U = 6863.50, *p* > 0.05.

### 3.4. Mental Adjustment to the Disease

Women suffering from ovarian, endometrial and breast cancer obtained the highest result of the Mini-Mac test in the areas of fighting spirit (M = 22.94, SD = 3.62) and positive reevaluation (M = 21.85, SD = 3.14) and the lowest in the area of helplessness–hopelessness (M = 12.43, SD = 4.34) (Table 7). These results were not differentiated by cancer location (in all areas *p* > 0.05).

Age correlates negatively with the average of the patients in the area of anxiety, ρ = −0.107, *p* < 0.05, which means that the younger patients attribute a higher average to this area. In turn, the results obtained in the area of fighting spirit, ρ = 0.220, *p* < 0.01, and positive reevaluation, ρ = 0.292, *p* < 0.01, correlate positively with age, so older patients attribute higher values to these areas.

Education affects mental adjustment to cancer in the areas of fighting spirit χ^2^(2) = 6.05, *p* = 0.049, hopelessness–hopelessness, χ^2^(2) = 7.28, *p* = 0.026 and positive reevaluation, χ^2^(2) = 9.66, *p* = 0.008, and for each of these areas, its average value decreases with an increasing level of education (Table 8).

The place of residence affects the areas of anxiety, U = 24,986.50, *p* = 0.021, and positive reevaluation, U = 24,205.00, *p* = 0.005. Women living in smaller towns (up to 100,000 inhabitants) scored higher in both these areas (M = 16.89, SD = 4.90 for anxiety; M = 22.23, SD = 3.07 for positive reevaluation) than women living in bigger towns (M = 15.89, SD = 4.46 for anxiety; M = 21.37, SD = 3.17 for positive reevaluation. Fighting spirit and positive reevaluation were strategies differentiated by income (U = 24,986.50, *p* = 0.002 for fighting spirit; U = 24,205.00, *p* = 0.001 for positive reevaluation) and professional status (U = 19,037.50, *p* = 0.002 for fighting spirit; U = 16,550.00, *p* = 0.001 for positive reevaluation). Women with higher income per family member (above PLN 1.500 net per month) obtained higher values in the area of fighting spirit (M = 23.12, SD = 3.74) and positive reevaluation (M = 22.20, SD = 3.03) than women with lower income (M = 22.79, SD = 3.52 for fighting spirit; M = 21.56, SD = 3.21 for positive reevaluation). Higher results in these two areas were obtained by patients who were pensioners (M = 23.31, SD = 3.90 for fighting spirit; M = 22.68, SD = 3.05 for positive reevaluation) than by patients who were professionally active (M = 22.65, SD = 3.17 for fighting spirit; M = 21.14, SD = 3.08 for positive reevaluation).

The diagnosis of metastases influenced the average results in the area of anxiety, U = 14,847.50, *p* = 0.007, and helplessness–hopelessness, U = 17,425.50, *p* = 0.002, in both cases these averages were higher in women with metastases diagnosed (M = 17.17, SD = 4.75 for anxiety; M = 13.40, SD = 4.88 for helplessness–hopelessness) than in women without metastases (M = 15.87, SD = 4.61 for anxiety; M = 11.89, SD = 4.08 for helplessness–hopelessness).

Chemotherapy treatment had no impact on the type of mental adjustment to the disease among the studied group of patients, (all comparisons, *p* > 0.05), radiotherapy treatment affected the results obtained in the areas of anxiety, U = 8884.50, *p* = 0.042, and helplessness–hopelessness, U = 7658.50, *p* = 0.001, and targeted treatment affected the area of hopelessness–hopelessness, U = 6189.00, *p* = 0.023. Patients treated with radiotherapy or targeted treatment attributed higher values to anxiety and helplessness–hopelessness (M = 14.18, SD = 4.17 in the group with radiotherapy in comparison with M = 12.22, SD = 4.32 in the group without radiotherapy; M = 13.97, SD = 4.50 in the group with targeted treatment in comparison with M = 12.30, SD = 4.31 in the group without targeted treatment).

## 4. Discussion

Strategies for coping with pain, acceptance of the disease and adjustment to cancer are important aspects of treatment and preservation of mental health. Socio-economic factors namely sex, age, education, income can influence treatment methods and results of psychometric tests measuring aspects of coping with the disease.

Therapeutic methods used in oncology are associated with anxiety of patients, including anxiety caused by the expected side effects [11,12]. With the commencement of oncological treatment, emotions experienced by patients vary [13,14]. A frequent result of the diagnosis of malignant neoplasms is increased stress, which can lead to mental health disorders such as anxiety and depressive reactions. One of the most common psychiatric disorders in oncology is depression [15,16], which occurs 1.5 times higher in women than in men [17,18]. In addition, in the aspect of gynecological diseases, cancer and its treatment affect the sexual and reproductive health of women and hamper psychophysical functioning as well as social relations [19]. In this context, the individual approach of patients to cancer, methods of pain control, strategies for coping with pain, and acceptance of the disease are of great importance.

Patients suffering from ovarian, endometrial and breast cancer in the author’s study most often controlled pain through the influence of physicians (M = 16.45, SD = 4.30). This is in line with the results of other studies where the physician figure is seen as the most important in pain management [20]. The method of pain control depended on age, place of residence, professional status, income, while the most diversified areas were the influence of physicians and random events.

The studied group of patients most often dealt with pain through praying/hoping (M = 21.47, SD = 8.89). According to other studies praying appears high in pain management strategies [21,22]. The studied socio-economic variables, but also the type of treatment used in patients influenced the patients’ strategies of coping with pain, mainly in the area of praying/hoping.

An important factor in the treatment of cancer is its acceptance [23]. In the author’s study, the average AIS acceptance score obtained by patients suffering from ovarian, endometrial and breast cancer was M = 27.48 (SD = 7.68). Higher education of patients, living in larger cities, and higher income per person in the household positively affected the acceptance of the disease by the patients. The findings are similar to other studies’ results [24,25]. The diagnosis of metastases and treatment with chemotherapy reduced the level of acceptance of the disease.

A study of patients with cancer, including breast, ovarian or cervical cancer, shows that the overall disease acceptance score was M = 25.35 (SD = 9.25). It was observed that the level of acceptance of cancer was dependent on the age of the patients [26].

Another study of patients with various cancers, including ovarian, cervical and breast, indicates that the average level of acceptance of the disease in these patients is M = 22.05 (SD = 9.41) [27], thus it was also lower in comparison to the average value obtained in the author’s study.

Among 140 women hospitalized at the gynecological department for diseases of the reproductive organs, the level of acceptance of the disease measured by the Approval Illness Scale was M = 28.15 (SD = 8.69) and analyzing the division of women into cancer patients and those suffering from non-oncological diseases, it turned out that women with cancer rated their quality of life worse (M = 25.37, SD = 8.06 and M = 29.38, SD = 8.71) [28].

The study conducted by Dryhinicz M. and Rzepa T. indicates even greater differences between the level of acceptance of the disease by patients diagnosed with cancer and other non-oncological diseases, for which the results are M = 20.68 (SD = 8.74) and M = 32.22, (SD = 9.44) respectively. There were statistically significant differences (*p* < 0.02) in the acceptance of the disease by oncological patients, with younger patients more accepting than older ones [29].

Acceptance of the disease is assessed relatively highly by women with breast cancer who have had mastectomy. These patients obtained the result of M = 28.60 (SD = 8.47) in the AIS test [30].

In another study of women with breast cancer after surgery, the average level of acceptance of the disease was M = 25.82, and the highest value of acceptance of the disease was obtained by women with lower education (primary or secondary) (*p* = 0.05) [31].

Adjustment to cancer is based on coping with the disease itself and its consequences, and in the long term on the necessary adjustment to the broadly understood changes in the quality of life. Patients after diagnosis are characterized by two attitudes. The active attitude is characterized by the fight for one’s health and life and general mobilization. In turn, the passive attitude manifests itself in fear and resignation. Attitude towards the disease significantly affects the quality of life of the patient, and most importantly, it may determine the long-term effects of treatment [32].

Patients suffering from ovarian, endometrial and breast cancer in the author’s study obtained the highest result of the Mini-Mac test in the areas of fighting spirit (M = 22.94, SD = 3.62) and positive reevaluation (M = 21.85, SD = 3.14). The areas of fighting spirit and positive reevaluation were differentiated by the majority of the socio-economic factors studied, and the type of treatment affected the level of anxiety and helplessness–hopelessness in patients.

The study involving patients with cancer, including ovarian, cervical and breast cancer using the Mini-MAC questionnaire, indicates that for individual areas of the test the results are as follows: fighting spirit M = 18.05; SD = 6.16, anxiety M = 18.51; SD = 5.65, helplessness–hopelessness M = 16.03; SD = 5.53, positive reevaluation M = 20.29; SD = 6.20 [28].

The study of women with cancer of reproductive organs and breast cancer does not show significant differences between the two groups in the individual areas of the Mini-MAC test [18]. Similarly, the study conducted by Szczepańska-Gieracha et al. among patients with breast cancer and cancer of the reproductive organs, after surgery and hospitalized due to cancer does not indicate differences between the style of adjustment to cancer by the studied groups of patients (for all areas of the Mini-MAC test *p* > 0.05) [33].

The study of women treated surgically for breast cancer who after mastectomy had an attitude of fighting spirit was characterized by a higher survival rate and absence of disease both after 5 and 10 years of diagnosis in comparison with women adopting the attitude of helplessness/hopelessness [18,34]. The results of another study of patients diagnosed with breast cancer indicate that both women after amputations and after conservative treatments preferred to deal with cancer in a constructive way [35].

The study conducted by Rogala D. et al. among women with cancer of reproductive organs hospitalized after hysterectomy indicates that constructive attitudes were dominant in the study group: positive reevaluation (M = 22.97, SD = 2.77) and fighting spirit (M = 22.95, SD = 3.25). The way of adapting to the disease of these patients was affected by marital status and material conditions [32].

Among women with cervical cancer, constructive strategies prevail, fighting spirit (M = 22.63, SD = 2.88) and positive reevaluation (M = 21.10, SD = 2.64). The result for anxiety was M = 16.07; SD = 4.42, and helplessness–hopelessness M = 12.63; SD = 3.76. In this case, women living in relationships also achieve, on average, higher scores in the application of helplessness–hopelessness strategies than single women (the result is also higher for women in relationships in the area of positive reevaluation) [36].

Analysis of the relationship between the coping strategy and the quality of life among women with cancer, mainly with breast cancer and cervical cancer, who underwent radiotherapy, showed that with an increased intensity of anxiety, the level of social functioning decreases, while in the case of an increased intensity of helplessness–hopelessness, the level of professional, cognitive, social functioning and general quality of life are reduced [37]. Similar results, indicating a large share of cognitive factors (the intensity of catastrophic thoughts) in experiencing symptoms (especially fatigue) is indicated by Jacobsen P. et al. [38].

## 5. Conclusions

To conclude, our study suggests that in terms of pain control, women with ovarian cancer, endometrial cancer, and breast cancer, attribute the highest importance to the influence of physicians. The influence of doctors and random events are the areas with the strongest differentiation by socio-economic factors. The most often selected strategy for coping with pain is praying/hoping, and this strategy is differentiated by all analyzed variables apart from the professional status.

The highest average disease acceptance score characterizes patients with breast cancer. Patients’ education, place of residence, income, as well as diagnosis of metastases and the type of treatment have an impact on the acceptance of the disease.

In the area of mental adjustment to the disease, patients are characterized primarily by fighting spirit, although all socio-economic variables affect the type of mental adjustment to cancer by the studied patients.

The awareness of the level of disease acceptance and pain management strategies of patients with cancer allows appropriate psychological support to be designed for specific groups of patients. In addition, knowledge of the impact of individual socio-economic factors on the attitudes of patients enables the concentration of this support in those groups of patients who need psychological support the most. This type of support may help change their attitude towards the disease. Further studies with the use of different methods are needed to fully understand patients’ adjustment to pain.

### Study Limitations

This study has limitations. In this study, no time measurement tools were used from the diagnosis of the disease until the date of this study. Although the time of the patient’s disease may influence the obtained results, the analysis of research carried out in this area does not explicitly confirm such relationships. The main limitation of our study was the lack of pain measurement in patients. Although pain is a subjective experience of patients, this factor could have influenced the results obtained in the psychometric tests.

## Figures and Tables

**Table 1:** Socio-economic characteristics of the sample.

	BPCQ Test Area	Total		Ovarian		Endometrial		Breast	
		*n*	%	*n*	%	*n*	%	*n*	%
Education	Primary/vocational	102	21.2	40	23.3	28	24.1	34	17.6
	Secondary	211	43.9	65	37.8	57	49.1	89	46.1
	Higher	168	34.9	67	39.0	31	26.7	70	36.3
Place	Up to 100,000 inhabitants	271	56.3	95	55.2	75	64.7	101	52.3
of Residence	More than 100,000 inhabitants	210	43.7	77	44.8	41	35.3	92	47.7
Professionally active	Yes	229	47.6	89	51.7	48	41.4	92	47.7
	No	252	52.4	83	48.3	68	58.6	101	52.3
Income	Up to 1500 PLN	221	45.9	68	39.5	54	46.6	99	51.3
	Less than 1500 PLN	260	54.1	104	60.5	62	53.4	94	48.7

BPCQ = Beliefs about Pain Control Questionnaire; *n* = sample size; % = percentage of sample.

**Table 2:** BPCQ Questionnaire results for patients with ovarian, endometrial and breast cancer.

BPCQ Test Area	Total		Ovarian		Endometrial		Breast		χ^2^	df	*p*
	M	SD	M	SD	M	SD	M	SD			
External factors	15.86	5.28	15.77	5.17	15.55	5.47	16.11	5.28	0.89	2	0.640
Influence of physicians	16.45	4.30	15.73	3.99	16.44	4.42	17.09	4.40	9.76	2	0.008
Random events	15.25	4.46	14.41	4.49	14.78	4.33	16.27	4.32	16.22	2	0.001

BPCQ = Beliefs about Pain Control Questionnaire; M = mean; SD = standard deviation; χ^2^ = Kruskal-Wallis’ H test value; df = degrees of freedom; *p* = significance.

**Table 3:** BPCQ test results for patients with primary, secondary, and higher education.

	Education					
BPCQ Test Area	Primary		Secondary		Higher	
	M	SD	M	SD	M	SD
External factors	16.88	6.00	16.36	5.17	14.60	4.72
Influence of physicians	17.75	3.87	16.86	4.29	15.15	4.24
Random events	16.27	4.58	15.80	4.16	13.93	4.46

BPCQ = Beliefs about Pain Control Questionnaire; M = mean; SD = standard deviation.

**Table 4:** CSQ test results for patients with ovarian, endometrial and breast cancer.

CSQ Test Areas	Total		Ovarian		Endometrial		Breast		χ^2^	df	*p*
	M	SD	M	SD	M	SD	M	SD			
Distraction	19.73	8.65	21.05	7.52	19.42	8.96	18.73	9.26	5.21	2	0.074
Reevaluation of pain sensations	12.57	9.13	13.28	8.96	12.63	8.59	11.89	9.58	2.87	2	0.239
Catastrophizing	11.67	8.04	12.22	7.86	12.65	8.47	10.60	7.83	5.99	2	0.050
Ignoring sensations	15.33	9.50	15.17	9.01	14.50	9.59	15.97	9.88	0.90	2.478	0.406
Praying/hoping	21.47	8.89	22.73	7.87	21.65	9.70	20.24	9.11	6.46	2	0.040
Declaring coping	21.39	9.21	21.60	9.10	20.38	9.18	21.81	9.32	1.67	2	0.434
Increased behavioral activity	20.86	8.68	21.20	8.22	20.31	9.58	20.90	8.53	0.37	2.478	0.692

CSQ = Pain Coping Strategies Questionnaire; M = mean; SD = standard deviation; χ^2^ = Kruskal-Wallis’ H test value; df = degrees of freedom; *p* = significance.

**Table 5:** CSQ test results for patients with primary, secondary, and higher education.

	Education					
CSQ Test Area	Primary		Secondary		Higher	
	M	SD	M	SD	M	SD
Distraction	20.99	8.28	20.33	8.46	18.21	8.93
Reevaluation of pain sensations	14.75	9.92	13.45	8.83	10.13	8.49
Catastrophizing	12.90	8.59	12.00	7.84	10.51	7.83
Ignoring sensations	17.71	9.78	16.30	9.17	12.66	9.18
Praying/hoping	24.05	8.74	21.64	8.99	19.68	8.47
Declaring coping	22.68	9.60	21.67	9.08	20.26	9.06
Increased behavioral activity	21.92	8.71	21.37	8.19	19.59	9.14

CSQ = Pain Coping Strategies Questionnaire; M = mean; SD = standard deviation.

**Table 6:** AIS Questionnaire results among patients with ovarian, endometrial and breast cancer.

AIS Test Area	Total		Ovarian		Endometrial		Breast	
	M	SD	M	SD	M	SD	M	SD
Acceptance of the disease	27.48	7.68	23.13	7.84	24.02	7.69	28.46	7.98

AIS = Approval Illness Scale; M = mean; SD = standard deviation.

**Table 7:** Mini-Mac test results for patients with ovarian, endometrial and breast cancer.

Mini-Mac Test Area	Total		Ovarian		Endometrial		Breast		χ^2^	*p*
	M	SD	M	SD	M	SD	M	SD		
Anxiety	16.45	4.73	16.65	4.15	17.06	5.07	15.91	4.97	4.07	0.131
Fighting spirit	22.94	3.62	22.42	3.91	22.90	3.74	23.43	3.21	5.65	0.059
Helplessness–hopelessness	12.43	4.34	12.84	4.38	12.71	4.59	11.89	4.10	5.01	0.082
Positive reevaluation	21.85	3.14	21.76	3.34	21.67	2.92	22.05	3.09	2.28	0.320

Mini-Mac = Mental Adjustment to Cancer; M = mean; SD = standard deviation; *p* = significance.

**Table 8:** Mini-Mac test results for patients with primary, secondary, and higher education.

	Education					
Mini-Mac Test Area	Primary		Secondary		Higher	
	M	SD	M	SD	M	SD
Anxiety	16.87	4.33	16.42	4.97	16.24	4.68
Fighting spirit	23.49	3.75	22.99	3.69	22.55	3.42
Helplessness–hopelessness	13.24	4.20	12.35	4.29	12.04	4.44
Positive reevaluation	22.46	3.09	22.04	3.04	21.26	3.21

Mini-Mac = Mental Adjustment to Cancer; M = mean; SD = standard deviation.

## Data Availability

The data analyzed during the current study are available.

## Abbreviations

BPCQ	Beliefs about Pain Control Questionnaire
CSQ	Pain Coping Strategies Questionnaire
AIS	Approval Illness Scale
Mini-MAC	Mental Adjustment to Cancer
M	mean
SD	standard deviation
*p*	probability value
χ^2^	Kruskal–Wallis H test
U	Mann–Whitney U test
ρ	Spearman’s correlation

## References

[1] Wojciechowska U., Czaderny K., Ciuba A., Olasek P., Didkowska J. (2018). Malignancies in Poland in 2016.

[2] International Association for the Study of Pain IASP Taxonomy. http://www.iasp-pain.org/Education/Content.aspx?ItemNumber=1698&navItemNumber=576#Pain(term:Pain).

[3] Nersesyan H., Slavin K.V. (2000). Current approach to cancer pain management: Availability and implications of different treatment options. Ther. Clin. Risk Manag..

[4] Whoqol Group (1995). The World Health Organization Quality of Life assessment (WHOQOL): Position paper from the World Health Organization. Soc. Sci. Med..

[5] Połocka-Molińska M., Jurczyk M. (2011). Quality of life of women with inoperable ovarian cancer. Curr. Gynecol. Oncol..

[6] Mangone L., Mandato V.D., Gandolfi R., Tromellini C., Abrate M. (2014). The impact of epithelial ovarian cancer diagnosis on women’s life: A qualitative study. Eur. J. Gynaecol. Oncol..

[7] Roland K.B., Rodriguez J.L., Patterson J.R., Trivers K.F. (2013). A literature review of the social and psychological needs of ovarian cancer survivors. Psycho-Oncology.

[8] Skevington S.M. (1990). A standardised scale to measure beliefs about controlling pain (B.P.C.Q.): A preliminary study. Psychol. Health.

[9] Juczyński Z. (2001). Measurement Tools in Health Promotion and Psychology.

[10] Rosenstiel A.K., Keefe F.J. (1983). The use of coping strategies in chronic low back pain patients: Relationship to patient characteristics and current adjustment. Pain.

[11] Bowers L., Boyle D.A. (2003). Depression in Patients With Advanced Cancer. Clin. J. Oncol. Nurs..

[12] Kyranou M., Paul S.M., Dunn L.B., Puntillo K., Aouizerat B.E., Abrams G., Hamolsky D., West C., Neuhaus J., Cooper B. (2013). Differences in depression, anxiety, and quality of life between women with and without breast pain prior to breast cancer surgery. Eur. J. Oncol. Nurs..

[13] Oudsten B.L.D., Van Heck G.L., Van der Steeg A.F.W., Roukema J.A., De Vries J. (2009). Clinical factors are not the best predictors of quality of sexual life and sexual functioning in women with early stage breast cancer. Psychooncology.

[14] Sucala M., Schnur J., Greene P., David D., Erblich J., Montgomery G. (2013). Cognitive-emotional equation: The relationship between irrational cognitive processes, cognitive contents, and specific emotions. Evidence from a sample of breast cancer patients. J. Cogn. Behav. Psychoter..

[15] Derogatis L.R., Morrow G.R., Fetting J., Penman D., Piasetsky S., Schmale A.M., Henrichs M., Carnicke C.L. (1983). The prevalence of psychiatric disorders among cancer patients. JAMA.

[16] Caruso R., Breitbart W. (2020). Mental health care in oncology. Contemporary perspective on the psychosocial burden of cancer and evidence-based interventions. Epidemiol. Psychiatr. Sci..

[17] Netzel P.J., Sampson M.S., Lapid I.M., Moore M.K., Rummans A.T. (2006). Depression and Anxiety in Cancer Patients a Focus on Breast and Gynecological Cancer.

[18] Malicka I., Szczepańska J., Anioł K., Rymaszewska J. (2009). Mood disorders and strategies for adapting to the disease in women treated surgically for breast cancer and cancer of reproductive organs. Contemp. Oncol..

[19] Enzlin P., De Clippeleir I. (2011). The emerging field of “oncosexology”: Recognizing the importance of addressing sexuality in oncology. Belg. J. Med. Oncol..

[20] Misterska E., Jankowski R., Głowacki M. (2013). Chronic pain coping styles in patients with herniated lumbar discs and coexisting spondylotic changes treated surgically: Considering clinical pain characteristics, degenerative changes, disability, mood disturbances, and beliefs about pain control. Med. Sci. Monit..

[21] Sullivan M.J.L., Thorn B., Haythornthwaite J.A., Keefe F., Martin M., Bradley L., Lefebvre J.C. (2001). Theoretical Perspectives on the Relation Between Catastrophizing and Pain. Clin. J. Pain.

[22] Utne I., Miaskowski C., Bjordal K., Cooper B.A., Valeberg B.T., Rustøen T. (2009). Confirmatory Factor Analysis of the Coping Strategies Questionnaire-Revised in Samples of Oncology Outpatients and Inpatients with Pain. Clin. J. Pain.

[23] Nowicki A., Ostrowska Ż. (2008). Acceptance of the disease by patients after breast cancer surgery during complementary treatment. Pol. Merkur. Lek..

[24] McCracken L., Eccleston C. (2006). A comparison of the relative of coping and acceptance-based measures in a sample of chronic pain sufferers. Eur. J. Pain.

[25] Perrot S., Poiraudeau S., Kabir M., Bertin P., Sichere P., Serrie A., Rannou F. (2008). Active or passive pain coping strategies in hip and knee osteoarthritis? results of a national survey of 4719 patients in a primary care setting. Arthritis Rheum..

[26] Kołpa M., Wywrot-Kozłowska B., Jurkiewicz B., Grochowska A. (2015). The factors determining acceptance and adjustment to cancer. Pielęgniarstwo Chir. Angiol..

[27] Ruszkiewicz M., Kreft K. (2017). Correlates of the acceptance of illness in a group of cancer patients. Psychoonkologia.

[28] Kaźmierczak M., Gebuza G., Izdepska E.K., Mieczkowska E., Gierszewska M. (2018). Influence of selected sociodemographic, obstetric and psychological factors on the level of acceptance of illness in women treated for reproductive system diseases. Nurs. Public Health.

[29] Dryhinicz M., Rzepa T. (2018). The Level of Anxiety, Acceptance of Disease and Strategy of Coping with Stress in Patients Oncological and Non-oncological. Ann. Univ. Mariae Curie-Skłodowska.

[30] Guil R., Ruiz-González P., Merchán-Clavellino A., Morales-Sánchez L., Zayas A., Gómez-Molinero R. (2020). Breast Cancer and Resilience: The Controversial Role of Perceived Emotional Intelligence. Front. Psychol..

[31] Łuczyk M., Pietraszek A., Łuczyk R., Stanisławek A., Szadowska-Szlachetka Z., Charzyńska-Gula M. (2015). Illness acceptance among women who have undergone surgical treatment for a breast neoplasm. J. Educ. Health Sport.

[32] Rogala D., Mazur A., Maślińska M., Koper K., Wysocka J. (2015). Self-efficacy and strategies of adaptation to disease in patients with cancer of reproductive organs. Curr. Gynecol. Oncol..

[33] Szczepańska-Gieracha J., Malicka I., Rymaszewska J., Woźniewski M. (2010). Psychological adjustment of women after oncological surgery and at the end of treatment. Współcz. Onkol..

[34] Brandão T., Schulz M., Matos P. (2017). Psychological adjustment after breast cancer: A systematic review of longitudinal studies. Psychooncology.

[35] Sobieralska-Michalak K., Kowalska J., Tudorowska M. (2016). The type of surgery and anxiety, depression, and adjustment to the disease in women diagnosed with breast cancer. Polish Psychol. Forum.

[36] Rogala D., Mazur A., Maślińska M., Krawczak M. (2016). Przystosowanie do choroby nowotworowej u pacjentek z rakiem szyjki macicy. Pielęgniarstwo Pol..

[37] Faller H., Olshausen B., Flentje M. (2003). Emotional distress and needs for psychosocial support among breast cancer patients at start of radiotherapy. Psychother. Psychosom. Med. Psychol..

[38] Jacobsen P., Andrykowski M.A., Thors C.L. (2004). Relationship of catastrophizing to fatigue among women receiving treatment for breast cancer. J. Consult. Clin. Psychol..

